# Analysis of promoter activity of members of the *PECTATE LYASE-LIKE (PLL) *gene family in cell separation in Arabidopsis

**DOI:** 10.1186/1471-2229-10-152

**Published:** 2010-07-22

**Authors:** Lingxia Sun, Steven van Nocker

**Affiliations:** 1 Department of Horticulture, Michigan State University, East Lansing, MI, USA

## Abstract

**Background:**

Pectate lyases depolymerize pectins by catalyzing the eliminative cleavage of α-1,4-linked galacturonic acid. Pectate lyase-like (*PLL*) genes make up among the largest and most complex families in plants, but their cellular and organismal roles have not been well characterized, and the activity of these genes has been assessed only at the level of entire organs or plant parts, potentially obscuring important sub-organ or cell-type-specific activities. As a first step to understand the potential functional diversity of *PLL *genes in plants and specificity of individual genes, we utilized a reporter gene approach to document the spatial and temporal promoter activity for 23 of the 26 members of the *Arabidopsis thaliana *(Arabidopsis) *PLL *gene family throughout development, focusing on processes involving cell separation.

**Results:**

Numerous *PLL *promoters directed activity in localized domains programmed for cell separation, such as the abscission zones of the sepal, petal, stamen, and seed, as well as the fruit dehiscence zone. Several drove activity in cell types expected to facilitate separation, including the style and root endodermal and cortical layers during lateral root emergence. However, *PLL *promoters were active in domains not obviously programmed for separation, including the stipule, hydathode and root axis. Nearly all *PLL *promoters showed extensive overlap of activity in most of the regions analyzed.

**Conclusions:**

Our results document potential for involvement of *PLL *genes in numerous aspects of growth and development both dependent and independent of cell separation. Although the complexity of the *PLL *gene family allows for enormous potential for gene specialization through spatial or temporal regulation, the high degree of overlap of activity among the *PLL *promoters suggests extensive redundancy. Alternatively, functional specialization might be determined at the post-transcriptional or protein level.

## Background

Pectins, a class of polysaccharide polymer typically characterized by a linear backbone of α-1,4-linked galacturonic acid (GalA) residues, are a major component of primary plant cell walls, and within the wall form a matrix in which a network of cellulose and hemicellulose is embedded [1-3]. Pectins contribute to the mechanical strength and physical properties of primary cell walls, but also function in intercellular adhesion [4-6], and can act as signaling molecules in morphogenesis and pathogen defense [2].

Plant growth and development is accompanied by dynamic remodeling of the cell wall, which in turn requires modifications of the various cell wall components including pectin. In accordance with the often complex structure of some pectins, an assortment of pectinase activities modify or degrade these polymers. Pectinesterases target methyl-esterified homogalacturonan, yielding substrates for polygalacturonases and pectate lyases, which cleave the GalA backbone [6]. Rhamnogalacturonases and rhamnogalacturonan lyases depolymerize branched regions of rhamnogalacturonan, whereas β-galactosidases and α-arabinosidases can degrade the galactan/arabinan/arabinogalactan side chains. Pectate lyases (EC 4.2.2.2) have been most extensively studied in *Erwinia chrysanthemi*, a major causal agent of soft-rot diseases that affect a wide range of plant species [7-9]. Their action not only results in maceration of plant tissues but also can activate plant defense systems [10-12]. Plant *PECTATE LYASE-LIKE *(*PLL*) genes encode proteins with strong amino acid sequence homology with the PelC isoform of bacterial pectate lyases [13,14] and exist as large families in plants where studied [15]. The abundance of *PLL *genes in plants, including 26 in Arabidopsis, 12 in rice, and 22 in poplar, has arisen from multiple gene duplication events [16,17], a process that may enhance plasticity in adaptation to changing environments [18]. Models predict that gene redundancy is evolutionarily stable only when duplicated genes differentiate in some aspect of their function, suggesting that individual members of large families such as *PLL *may have some unique function [18].

Various analyses of *PLL *genes from plants revealed expression in a broad range of organs and plant parts including root, leaf, flower, pollen, filament, style, pistil, and ripening fruit [14,16,19-24]. *PLL *genes in several studies also showed elevated expression in response to auxin, wounding, and/or pathogen infection [16,25-28]. Transcriptional analysis in Arabidopsis revealed that a small subset of *PLL *genes were up-regulated during stamen abscission or in cortical cell separation during the emergence of the lateral root [16,29,30]. Reduced expression of strawberry *PL1 *in transgenic strawberry plants suggested a natural role in tissue softening during fruit ripening [31]. All of these data implicate *PLL *genes in various plant growth and development events. However, the activity of these genes has been characterized only at the level of entire organs or plant parts, potentially obscuring important sub-organ or cell-type-specific activities.

During plant growth and development, there are many events in which adjacent cells separate in a coordinated manner. Cell separation resulting in organ abscission, or anther or fruit dehiscence, occurs in predetermined positions, called abscission zones (AZs) or dehiscence zones (DZs), respectively [32,33]. Intercellular space formed in leaves and stems can result from restricted separation of cells at the tricellular regions [34]. Fruit ripening also involves limited cell separation, in which only the middle lamella is degraded, with tricellular junctions and plasmodesmata often remaining intact [35,36]. Targeted cell separation is also involved in the processes of seed germination, lateral root emergence, pollen tube penetration through the transmitting tract, and shedding of columella root cap cells [37,38]. In this study, we examined spatial-temporal expression patterns of promoters of *PLL *family members, focusing on the various cell separation and wall loosening events that occur during Arabidopsis growth and development.

## Results and Discussion

### Phylogenetic analysis

**Figure 1:**
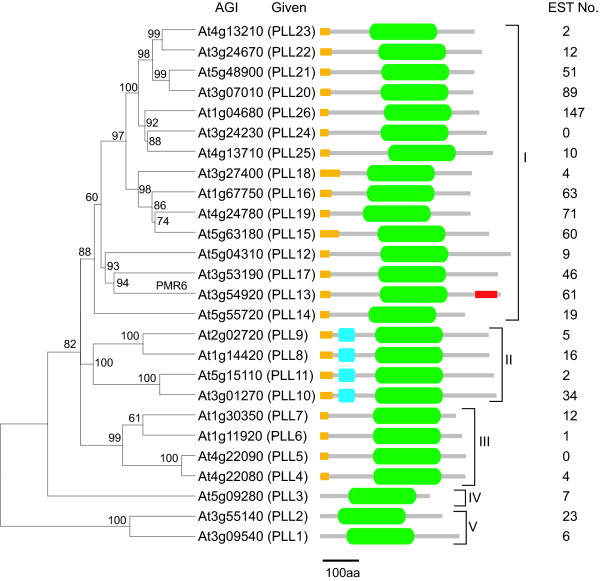
**Phylogenetic analysis of Arabidopsis PLL amino acid sequences**. For each gene, the Arabidopsis Genome Initiative (AGI) designation is given along with the *PLL *designation given in this study. The number of ESTs for each gene found in the NCBI database is indicated at right. The Pec_lyase_C domain is indicated as a green oval. The amino-terminal signal peptide is represented by a yellow rectangle. The Pec_lyase_N domain is indicated as a blue rectangle. The predicted carboxyl-terminal glycosyl-phosphatidylinositol (GPI) anchor site in PMR6 (PLL13) is indicated by a red rectangle. Bootstrap values are shown above nodes and indicate how consistently the data support the given taxon bipartition.

Based on peptide sequence homology with the enzymatically characterized pectate lyase PL1 from banana and annotated protein domains, the Arabidopsis genome encodes for 26 pectate-lyase-like proteins. Neighbor-joining analysis partitioned these protein sequences into five subfamilies (**Figure **1). This genomic content and phylogenetic organization is similar to that previously reported for *PLL*s from multiple plant species including Arabidopsis [16,21]. All Arabidopsis PLLs exhibited a region of sequence homology with pectate lyase C (pelC) from *Erwinia chrysanthemi*, annotated as the Pec_lyase_C (Pfam00544) domain (**Figure **1) and 23 of the proteins contain a probable amino-terminal signal peptide. The four members included in Subfamily II (PLL8-11) also exhibit a Pec_lyase_N (Pfam04431) domain, a short region of high homology whose structure or function has not been described. The predicted carboxyl-terminal glycosyl-phosphatidylinositol (GPI) anchor site previously identified in PMR6 (POWDERY MILDEW RESISTANCE 6, also called PLL13) was not obviously present in other PLLs, suggesting a specialized function of PMR6 associated with powdery mildew susceptibility [28] (**Figure **1).

### Estimation of *PLL *gene expression through analysis of public transcriptome data

As a first step to assess expression pattern of *PLL *genes in Arabidopsis, we analyzed publicly available transcriptome data. Expressed sequence tags (ESTs) found in public databanks corresponding to individual *PLL *gene family members were found to be sourced from various tissues and stages across Arabidopsis growth and development, as well as various environmental conditions (not shown). Representational frequency of ESTs for individual *PLL *genes was highly variable both across the gene family and within subfamilies (**Figure **1). Analysis of publicly available microarray data also suggested that there was at least some unique developmental pattern of expression for most *PLL *genes (see **Additional file **1). A striking exception are the four members of Subfamily II (*PLL8-PLL11*), which showed a similar expression pattern localized predominately to pollen and stamens (see **Additional file **1). Several genes (*PLL3*, *4*, *6*, *7*, *12*, and *24*) were transcriptionally silenced across most sampled tissues, whereas *PLL2 *was apparently expressed ubiquitously, suggesting a very general function (see **Additional file **1). These data are generally consistent with the results of qualitative RT-PCR analysis of *PLL *genes [16] and support a collectively ubiquitous function for *PLL*s in growth and development and the potential for functional specialization by many of these genes.

### Analysis of *PLL *promoter activity

Direct approaches for analysis of gene expression such as RNA-seq, microarray analysis and RT-PCR can be problematic when applied to large gene families, due to sequence homology among family members and/or inability to resolve expression at the cellular level. Accordingly, we utilized a histological reporter gene (*β-glucuronidase*, *GUS*) to determine the expression regulatory potential of individual *PLL *gene promoters. We engineered the *GUS *coding sequence adjacent to up to ~2 kb of 5' UTR/promoter sequence, preserving the authentic start codon of the *PLL *genes, and expressed the *PLL::GUS *fusions in transgenic Arabidopsis (see **Additional file **2). For each *PLL *gene, we analyzed at least four independent transgenic lines. GUS activity patterns were generally consistent between independent lines, with the exception of three (*PLL6*, *PLL12*, and *PLL14*) that showed weak and variable activity and were not analyzed further. No GUS activity was observed in transgenic plants transformed with a promoterless G*US *construction, or in non-transgenic plants, in any plant part or under any condition used for analysis. Based on the known roles for pectins in cell adhesion and cell wall architecture, we focused our analysis on developmental events closely associated with cell separation.

### *PLL::GUS *expression associated with cell separation

**Figure 2:**
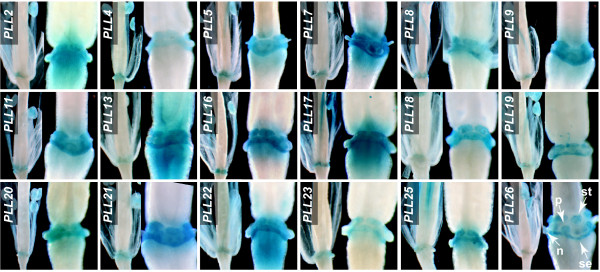
**Spatial and temporal *PLL::GUS *expression in the AZs of sepals, petals, and stamens**. Each frame is a composite of two independent photographs showing flowers at Stage 16 (left) and Stage 17 (right). Arrowheads in the *PLL26 *panel exemplify the location of AZs of the sepal (se), petal (pe), and stamen (st). A nectary (n) is indicated.

**Floral organ AZs**. Cell separation in Arabidopsis has been best characterized in the context of floral organ abscission [32]. Arabidopsis exhibits abscission of sepals, petals, and stamens following pollination. Abscission is conditioned by cell separation within the AZ, a tightly localized region at the base of the floral organs [39]. We analyzed developing flowers at various stages until Stage 18, when siliques began to yellow [40]. (**Figure **2 and see below). GUS activity within the AZs was observed for 18 *PLL *promoters (**Figure **2 and see **Additional file **3). For all of these, GUS activity was first detected in the AZs of sepals, petals, and stamens at Stage 16, which followed anthesis by about 2 days and was marked by the withering of sepals and petals (**Figure **2) [40]. GUS activity had increased within the AZs of all three organ types by Stage 17, when perianth organs were abscising (**Figure **2). For all *PLL *promoters, GUS activity in the AZs was detectable but very weak at Stage 18 (**not shown**). Activity for *PLL26 *in floral organ AZs was apparent through Stage 19, marked by valve separation (not shown). These results are consistent with those of Cai and Lashbrook [29], who identified *PLL18 *as substantially upregulated in the stamen AZ through transcriptional profiling of laser-capture-microdissected tissues. This upregulation was apparent in the latter part of Stage 15, following anthesis by approximately 36 h [29,40].

### Fruit DZ and seed AZ

**Figure 3:**
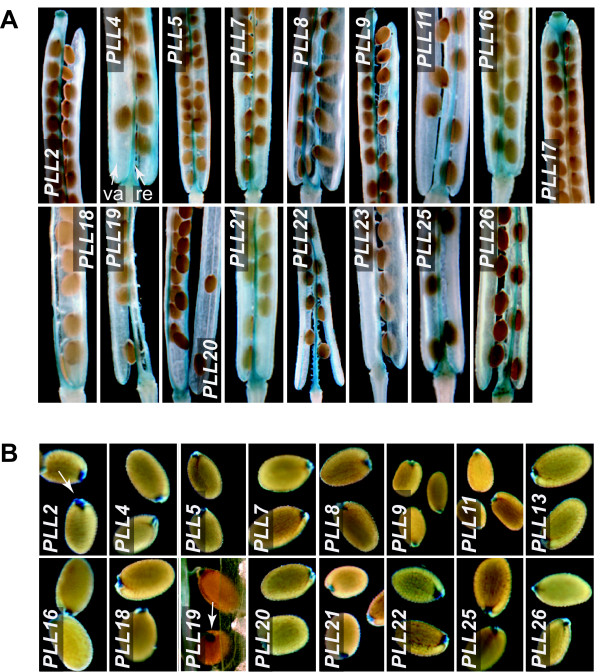
***PLL::GUS *expression in the silique DZ and seed AZ**. (A) Siliques at Stage 19, beginning to dehisce and showing GUS activity in the DZ. The silique valve (va) and replum (re) are indicated with arrowheads in the *PLL4 *panel. (B) Recently abscised, mature seeds showing GUS activity in the region of the seed AZ (arrowhead in *PLL2 *panel). The arrowhead in the *PLL19 *panel shows GUS activity in this region just before abscission.

The Arabidopsis fruit consists of two valves separated by a replum. Mature fruits dehisce due to cell separation in the so-called DZ, which is distributed along the valve margins and includes a separation layer [41]. Mature seeds are released from the funiculus, a stalk-like structure connecting seeds to the replum, at a site referred to as the seed AZ. We analyzed GUS activity in these regions during development of flowers and fruit from Stage 16 through Stage 20, marked by seed abscission [40] (**Figure **3A). We found that 17 of the *PLL *promoters drove GUS activity within the apparent DZ of developing siliques. This subset of *PLLs *nearly completely overlapped with the subset driving expression in floral organ AZs (see **Additional file **3). For all, GUS activity was evident at the onset of stage 18, which preceded separation of the valves by approximately 24 h (**not shown**). GUS activity was first seen at the basal and apical ends of siliques, where valve separation was initiated, and then became established along the entire length of the fruit as valve separation progressed (**Figure **3A and **not shown**).

A mostly overlapping subset of 16 *PLL *promoters drove GUS activity within the apparent seed AZ (**Figure **3B and see **Additional file **3). For all of these, GUS activity was not observed at the beginning of stage 19, but was obvious in seeds that were still attached to the funiculus and septum before the beginning of Stage 20 (e.g., *PLL19 *in **Figure **3B).

### Radicle emergence during seed germination

**Figure 4:**
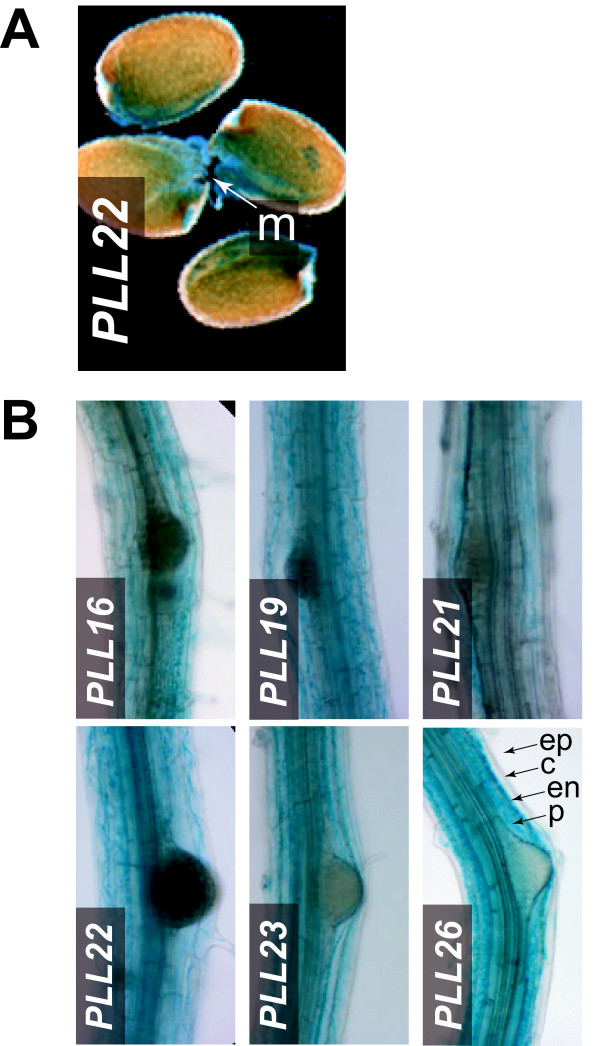
***PLL::GUS *expression in germinating seeds and during lateral root initiation**. (A) *PLL22 *activity at the micropylar (m) end of germinating seeds, during endosperm rupture and preceding radicle emergence. (B) *PLL::GUS *activity in the region of emerging lateral roots of 12-d-old plants. Arrowheads indicate epidermis (ep), cortex (c) endodermis (en), and pericycle (p) layers.

During seed germination in Arabidopsis, the radicle penetrates a single endosperm cell layer at the micropylar end of the seed [42]. To assess *PLL *promoter activity in this region, we stained seeds for GUS activity 24 h after imbibition, when approximately half of the seeds showed rupture of the testa but before radicle emergence was seen (**not shown**). Two *PLL *promoters (*PLL16 *and *PLL22*) directed GUS activity in germinating seeds; in both cases GUS activity was observed in the micropylar end before obvious emergence of the radicle (**Figure **4A and **not shown**).

### Lateral root initiation

Arabidopsis lateral roots initiate from the pericycle cell layer and must penetrate the overlaying endodermal, cortical and epidermal layers during emergence [43]. Although several *PLL *promoters drove GUS activity at relatively weak levels along the axis of primary roots (see below), six (*PLL16*, *19, 21-23, 26*) showed strong activity in primary roots of 12-d-old plants that were initiating lateral roots. This activity was confined to the endodermal and cortical layers in the region of initiation, but was not obviously restricted to cell layers directly overlaying the new lateral root primordia (**Figure **4B and **not shown**). We did not observe strong GUS staining in the epidermis for any of the six promoters (**Figure **4B and **not shown)**. These findings are consistent with the increased transcriptional accumulation previously reported for *PLL16 *and *PLL26 *during lateral root emergence induced by auxin [26,30].

### *PLL::GUS *expression in other developmental contexts

To analyze *PLL *promoter activity in a broader developmental context, including processes potentially independent of cell separation, we undertook a comprehensive analysis of the activity of all of the studied *PLL *promoters during growth and development of Arabidopsis.

### Seedlings

**Figure 5:**
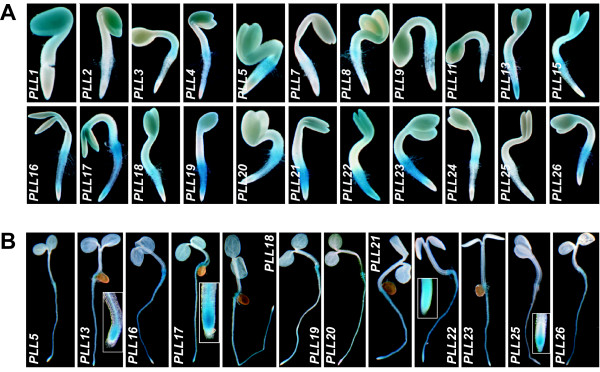
***PLL::GUS *expression in seedlings**. (A) GUS activity in 1-d-old seedlings. (B) GUS activity in 5-d-old seedlings. Inset boxes in the panels for *PLL13*, *PLL17*, *PLL22*, and *PLL25 *show root tips at higher magnification.

GUS activity was detected in 1-d-old seedlings for 22 *PLL *promoters. In seedlings, GUS staining was seen in the cotyledons, hyocotyl, root/hypocotyl junction, root axes, and/or root tips (**Figure **5A and see **Additional file **3). A subset of 11 of these promoters also drove GUS activity along the root axes in 5-d-old seedlings (**Figure **5B and see **Additional file **3); this subset included the six that were strongly active during lateral root initiation (above). Four promoters, *PLL13*, *PLL17*, *PLL22*, and *PLL25*, were active in the root tips*. PLL25 *was unique in that it drove GUS activity specifically in the root tip. Both *PLL22 *and *PLL25 *drove expression in a broad region that included the columella root cap (**Figure **5B).

### Hydathodes and Stipules

**Figure 6:**
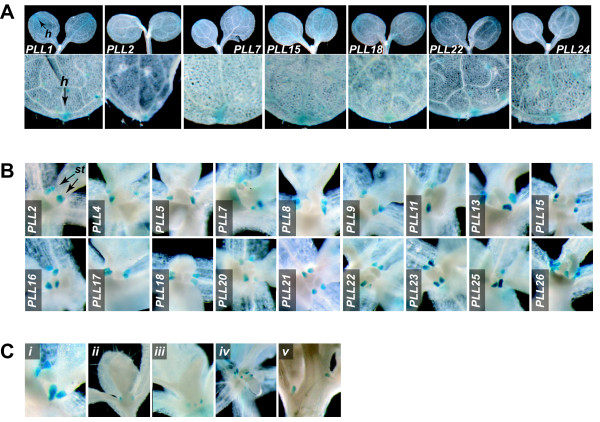
***PLL::GUS *expression in hydathodes and stipules**. (A) GUS activity in hydathodes. In the upper row, 5-d-old seedlings with fully expanded cotyledons are shown. In the lower row, the distal tips of the first rosette leaf on 8-d-old seedlings are shown. The position of the hydathode (h) is indicated for the *PLL1 *panels. (B) GUS activity in the stipules of an expanding leaf of 6-d-old seedlings. (C) GUS activity in stipules of *PLL19::GUS *plants at various stages of plant and leaf development: (i) expanding ~3 mm leaf of 6-d-old seedling; (ii) expanding ~6 mm leaf of 6-d-old seedling; (iii) nearly fully expanded leaf (~12 mm) of 8-d-old seedling; (iv) apex of 15-d-old plant showing staining in stipules of primordial cauline leaves; (v) flowering plant showing staining in the stipules of fully expanded cauline leaves

We observed GUS activity in fully expanded cotyledons and rosette leaves for seven *PLL *promoters (**Figure **6A). For all seven, activity was restricted to the tip of the cotyledon/leaf. This corresponds to the position of the hydathodes, specialized pore-like structures that release water and solutes from the xylem [44].

GUS activity was apparent within the shoot apex of plants in the vegetative phase for 19 *PLL *promoters. Upon close examination, in all cases this activity appeared in tightly localized foci bordering the base of initiating leaves, including cauline leaves (**Figure **6B**and not shown**), at the position of the stipules. In Arabidopsis, stipules are apparent on the newly formed leaves but become less obvious upon leaf expansion. For all *PLL*s, GUS activity was strongest in those leaves that had initiated and were undergoing expansion, and weakest or not apparent in fully expanded leaves (**Figure **6C**and not shown**). The function of stipules in Arabidopsis development remains largely unknown. However, it has been demonstrated that stipules are primary sites for the accumulation of auxin associated with vascular differentiation and leaf morphogenesis [45,46]. No GUS activity was observed for any *PLL *promoter in the apex after the transition to flowering (**not shown)**.

### Base of flower/fruit pedicel

**Figure 7:**
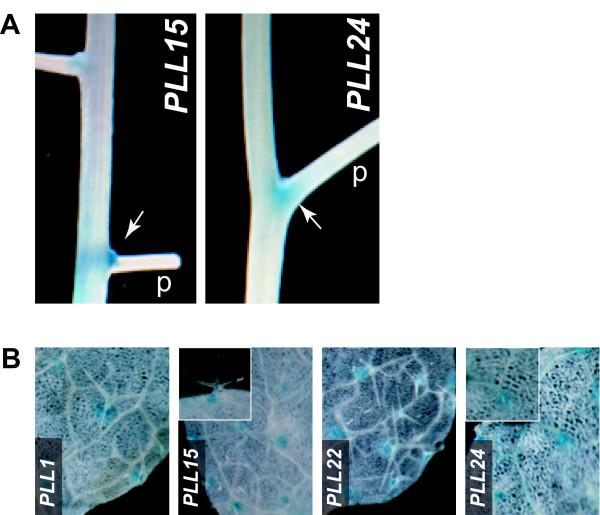
**GUS activity at the base of pedicels and trichomes**. (A) Segment of inflorescence of *PLL15::GUS *and *PLL24::GUS *plants showing staining (arrowheads) within the base of pedicels (p) of immature fruits. (B) Fully expanded leaves of 12-d-old plants showing GUS staining at the base of trichomes. Insets in the panels for *PLL15 *and *PLL24 *show enlarged views of individual trichomes.

Two *PLL *promoters (*PLL15 *and *PLL24*) drove GUS activity within a restricted region on the adaxial side of the base of pedicels (**Figure **7A). For both, this was apparent after Stage 12 of floral development, just before anthesis, and through development of the green silique (**not shown**). The *PLL15 *and *PLL24 *promoters, in addition to *PLL1 *and *PLL22*, also drove GUS activity at the base of trichomes on the leaf epidermis (**Figure **7B and see **Additional file **3). In all cases, GUS activity was limited to the stellate trichomes on the adaxial surface of rosette leaves, in the region roughly corresponding to basement cells, a group of specialized cells encircling the trichome and potentially serving as structural and/or biochemical support for the trichome (**Figure **7B).

### Flowers

**Figure 8:**
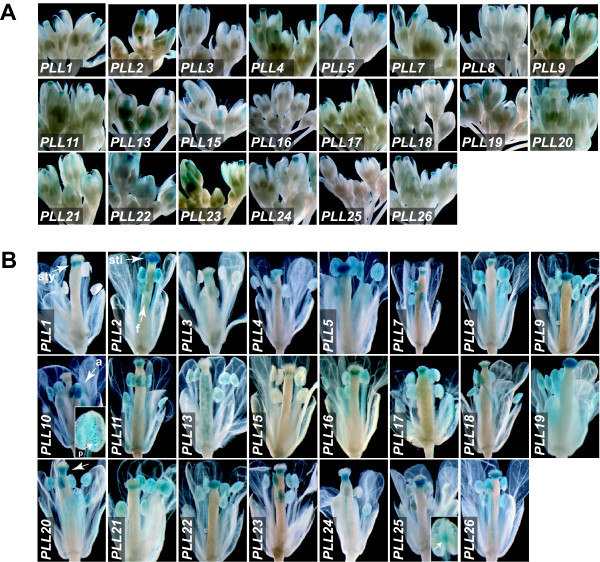
**PLL::GUS expression in various parts of the flower**. (A) Inflorescences of *PLL::GUS *plants showing flowers at various developmental stages through Stage 12. (B) Individual flowers from *PLL::GUS *plants at floral Stage 13. Arrowheads indicate style (*PLL1 *panel, sty), stigma (sti) and filament (f)(*PLL2 *panel), anther (a) and pollen (p)(*PLL10 *panel), staining distributed along the style and basal portion of the stigma (*PLL20 *panel), and staining at the junction of filament and anther (*PLL25 *panel, inset).

GUS activity was detected in developing flowers for all 23 of the *PLL *promoters (**Figure **8 and see **Additional file **3). None of the promoters studied showed strong activity before Stage 11, which is marked by appearance of stigmatic papillae [40] (**not shown)**. All except *PLL10 *drove GUS activity in varying domains within the style and/or stigma (**Figure **8A**and **8B and see **Additional file **3). Some of these showed localized expression in the style (e.g., *PLL1*), whereas others showed expression throughout the stigma (e.g., *PLL2*), or in the basal portion of the stigma extending into the style (e.g., *PLL20*). A potential function for *PLL *genes expressed in the stigma and style is to facilitate pollen tube growth, via separation of transmitting tract cells and/or remodeling of cell walls in this tissue. In the stamen, several promoters drove activity in the filament (e.g., *PLL2*), and/or throughout the anther and predominantly localized to pollen sacs and/or developing pollen (e.g., *PLL10*). The subset of *PLL*s driving expression in pollen is largely consistent with that determined based on transcriptome analysis (see **Additional file **1) or RT-PCR [16]. *PLL3 *and *PLL25 *were distinguished by driving GUS activity in the stamen limited to the junction of the filament and anther (**Figure **8B and see **Additional file **3). *PLL13 *drove expression in the style in immature flowers (**Figure **8A), but by Stage 15 this activity was not apparent, and instead GUS staining was seen in developing seeds (**Figure **8B). Activity of *PLL13 *was not seen in ovules at Stage 12 or earlier (**Figure **8A), or in developing seeds at Stage 16 or later (**Figure **2). We hypothesize that this seed-associated pattern is associated with the cellularization of the endosperm [47].

### Correlation or divergence of *PLL *promoter activity with patterns of RNA accumulation

To evaluate the potential for regulation of *PLL *genes outside of the context of promoter activity, we analyzed our findings in relation to previously reported transcriptional patterns based on microarray analysis of Arabidopsis organs or whole structures (see above and **Additional file **1[48].

Most notably, the strong promoter activity found in anthers for the four members of Subfamily II (*PLL8-PLL11*) (**Figure **8) corresponded well with the stamen-associated pattern of mRNA localization (see **Additional file **1). However, *PLL8, 9, and 11 *also showed strong promoter activity variously outside of flowers in the hypocotyl (*PLL11*), root/hypocotyl junction (*PLL8 *and *9*), and stipules (*PLL8 *and *11*), regions where transcriptional profiling did not obviously predict promoter activity. Genes expected to be transcriptionally quiescent based on microarray analysis, such as *PLL4 *or *PLL24*, showed promoter activity that was relatively strong, and that was similar to genes that showed strong transcriptional signals such as *PLL8 *or *PLL15*, respectively. *PLL2*, which might be expected to be constitutively expressed as a 'housekeeping' gene based on microarray analysis, showed promoter activity in only a subset of the regions analyzed (see **Additional file **3). At least part of these apparent inconsistencies between promoter activity analysis and transcriptional profiling can be attributed to our qualitative representation of promoter activity, which is in reality a quantitative function. The lack of high values for mRNA accumulation in structures (e.g., the silique) that include tissues with strong promoter activity (e.g., the DZ) could be simply explained by the fact that such tissues make up only a small portion of the bulk of the regions subject to analysis. Other cases where mRNA levels are low in spite of strong regional promoter activity might be attributed to rapid mRNA turnover. It is most difficult to explain the accumulation of mRNAs in tissues or structures that lack detectable promoter activity, for example the strong and ubiquitous mRNA expression of *PLL2*. In these cases regulatory elements required for appropriate expression might lay outside of the ~2 kb 5' region utilized to direct *GUS *expression.

## Conclusions

In this study we determined the temporal and regional promoter activity for members of the *PLL *gene family during growth and development of Arabidopsis. Given the ubiquitous role of pectins in intercellular adhesion and cell wall architecture, and the various organismal processes that require targeted cell separation or cell wall remodeling, these genes are expected to be of fundamental importance.

We found that, as expected, promoter activity was common in cell types programmed for abscission, most obviously the perianth floral organ AZs, the DZ of the fruit, and the seed AZ. Activity was also seen in regions, such as cell layers in the region of initiating lateral roots, where cell separation is expected but has not been well characterized. Pectate lyase activity is expected to act in concert with other cell wall remodeling activities both to condition cell separation and to modify newly exposed cells for barrier functions. Interestingly, we saw more limited *PLL *promoter activity at the base of pedicels of flowers and fruits. Cells in these regions can be artificially programmed to undergo separation in Arabidopsis, in response to ectopic activity of the small abscission-associated peptide IDA [49], suggesting an abscission-related role. If these *PLL *genes do have a cryptic function in flower/fruit abscission, abscission may be normally limited by the degree of pectate lyase activity or deficiency of other, cooperative, cell wall-modifying activities. Interestingly, the two *PLL *promoters driving activity in this region also were active in cells at the base of trichomes, suggesting their pedicel- and trichome-associated roles may be analogous. Both regions are subject to mechanical stress imparted by movement of the pedicels/trichomes and an alternative function for these *PLL*s may be to participate in a pathway of cell wall remodeling to accommodate such stress.

Other patterns of *PLL *activity were difficult to explain given a function limited to cell separation, and might instead be associated with general cell wall remodeling accompanying growth and differentiation. The extensive and various activities seen in the root is a good example of this. We can not provide a simple explanation for the strong and widespread promoter activity in stipules; however, these structures are absorbed into the leaf periphery during leaf expansion and this process could be accompanied by radical changes in cell wall morphology. Interestingly, both stipules and hydathodes were previously characterized as sites of high free auxin concentration during early leaf development [45]. A subset of *PLL *promoters in our study were found to be actively associated with lateral root emergence, or in the region of the root apical meristem, other sites associated with local auxin concentration maxima [30,50]. These observations suggest a close functional relationship between these *PLL*s and auxin.

We note that many promoter activities of the *PLL *genes are superficially similar to those exhibited collectively by several presumed polygalacturonase genes [51]. These include cases intuitively related to cell separation (perianth organ AZs, sites of lateral root emergence, fruit DZ, seed AZ, germinating seed) and cases in which activity may be limited to cell wall remodeling (developing pollen and seeds). This implies close functional association between PLLs and PGs, which is expected given that both should target pectins.

An obvious use of data from high-throughput analyses of promoter activity and/or transcriptional profiles is to guide reversed-genetics analyses, especially where phenotypes are difficult to anticipate and/or where potential redundancy of the targeted genes is a limitation. The original intent of this project was to identify individual or small subsets of *PLL *genes that might be targeted in this manner. However, the surprisingly high degree of overlap both in promoter activity and RNA accumulation observed among the *PLL *genes/promoters largely precluded this objective. We note that neither our promoter activity analysis nor previous microarray data would intuitively predict the documented non-redundant role for *PLL13 *(*PMR6*) in facilitating infection by powdery mildew [28]. This role is accomplished where infection occurs in leaf cells, where neither transcriptional profiling nor our promoter analysis suggested strong or non-redundant expression.

Another obvious application of transcriptional data is the computational identification of DNA regulatory elements linked to regionally or temporally specific expression patterns. Here, it is noteworthy that the correlation between the transcriptional profiles previously established for these genes [16,48] and their promoter activity (this study) was apparently very limited. This justifies the additional time and expense necessary to accomplish comprehensive promoter activity profiles, and/or cell-type specific transcriptional profiling to define transcriptional regulatory elements.

The *PLL *gene family is one of the largest and most complex in Arabidopsis, suggesting enormous potential for gene specialization through spatial or temporal regulation. However, we found a remarkably high degree of overlap of activity among the *PLL *promoters. The activity of 19 of the 23 tested *PLL*s in the stipules is an excellent example of this, as is the activity of at least 18 in the apparent AZs of the sepals, petals and stamens at similar stages of development. This observation is surprising given that gene duplications should be evolutionarily stable only after having acquired a useful, non-redundant function. For PLLs, functional specificity may be bestowed by post-transcriptional and downstream mechanisms. Such specificity might include regulation of RNA stability. Specificity might also be directed at the protein level, through potential differences in localization or mobility within the apoplast. Alternatively, given the expected dynamic pH environment of the apoplast, the proteins might function only within a narrow pH window. Finally, various PLL proteins might show preference for specific pectin substrates, an intriguing possibility given the structural complexity of pectins. Many of these possibilities could be addressed by the purification (or recombinant synthesis) and enzymatic characterization of individual PLLs.

## Methods

### Plant growth conditions

Wild type *Arabidopsis thaliana *(ecotype Col-0) was used for all experiments. Standard growth conditions were 16-h-cool white fluorescent light/8-h-dark photoperiods at 22°C. For growth in sterile culture, seeds were surface-sterilized and germinated on one-half-strength Murashige and Skoog (MS) medium supplemented with 10 mM MES [2-(N-morpholino) ethanesulfonic acid] (Sigma, St. Louis, MO), pH adjusted to 5.7, and solidified with 0.8% phytoagar (Invitrogen, Carlsbad, CA).

### Phylogenetic analysis and protein domain identification

The peptide sequence of an enzymatically confirmed pectate lyase from banana, *Musa acuminata PL1 *(GenBank accession AF206319) [22], was utilized as a query in BLASTP (Basic Local Alignment Search Tool Protein) analysis of Arabidopsis translated open reading frames [The Arabidopsis Information Resource (TAIR) Version 7] [52], and closely related proteins [Expect (E) value < 8E-36] were used for phylogenetic tree construction. Predicted amino acid sequences were aligned using ClustalW2 [53,54], and MEGA4 [55] was used to construct the phylogenetic tree. Annotated PLL protein domains were identified in the Pfam database [56,57] as follows: PF03211, Pectate_lyase; PF00544, Pec_lyase_C; PF09492, Pec_lyase; PF04431, Pec_lyase_N. Amino-terminal signal peptides were defined by SignalP 3.0 [58] using a maximum S-score larger than 0.95 and signal peptide probability greater than 0.80 [59]. The number of ESTs for each gene in the NCBI Unigene database was found at NCBI [60].

### Transgenic plant construction

To engineer *GUS *as a reporter for *PLL *promoter activity, up to approximately 2 kb of genomic DNA, upstream from the start codon, was amplified by PCR using gene-specific primers (see **Additional file **4) containing unique restriction sites. In general, where the upstream intergenic region was less than 2 kb, all of the region was included (see **Additional file **2). All amplification products were sequenced to confirm lack of PCR-induced mutation, and cloned into the pCAMBIA1305 vector (Cambia, Black Mountain, Australia) modified to contain an *Nco*I site at the start codon of the *GUS *gene and multiple cloning sites replacing the pCAMBIA Lac Z alpha and CaMV35S regions. This resulted in *PLL*::GUS fusions that preserve the authentic 5' UTR and start codon position. Plasmid DNAs were introduced into *Agrobacterium tumefaciens *strain GV3101 and transformed into wild-type plants using the floral dipping method [61]. Transgenic plants were subjected to selection with the herbicide glufosinate (Basta; Beyer CropScience, Barmen, Germany).

### Histochemical GUS assays

Populations derived from at least four independently transformed transgenic lines were analyzed for each *PLL::GUS *construction. For histochemical GUS assays, plants or plant parts at various developmental stages were immersed in GUS staining solution [0.5 mM X-gluc, 0.5% (v/v) Triton X-100, 50 mM sodium phosphate buffer (pH adjusted to 7.2)] under vacuum infiltration for 5 min, and incubated at 37°C for various lengths of time. After staining, tissues were incubated in 70% ethanol for several hours to remove chlorophyll. Staining was visualized using a Nikon dissecting microscope equipped with a digital camera. Flower developmental stages annotated in this study were described by Smyth et al [40].

### Analysis of AtGenExpress data

Microarray data were collected from the AtGenExpress Development data set [48]. Only results from wild-type plants were included in the analysis. The log_2_-transformed absolute signal values were used in hierarchical clustering based on average linkage (Cluster version 3; [62]), and results were visualized in TreeView [62].

## Authors' contributions

LS and SvN both participated in the planning and execution of the experiments, analysis of results, and writing of the manuscript.

## Supplementary Material

Additional file 1 **Hierarchical clustering of RNA accumulation for Arabidopsis *PLL *genes in various plant parts/developmental stages based on public microarray data**. Plant parts and growth stages are labeled according to AtGenExpress [48]. Microarray signal value is indicated by color from yellow (lowest) to red (highest). *PLL5 *was not represented on the microarray used in this analysis. Click here for file

Additional file 2 **Depiction of promoter region from individual *PEL *genes utilized to drive *GUS *expression**. Genes are aligned with the predicted translational start (ATG) codon at right. The scale of upstream distance (kb) is given at top. The portion of the upstream region used is indicated with a heavy line. Upstream features (mapped 5' or 3' RNA ends and/or predicted translational start/stop codons) delineating the intergenic region are indicated with relative position given at left. Click here for file

Additional file 3 **Summary of *PLL::GUS *expression in various Arabidopsis parts**. Click here for file

Additional file 4 **Primer sequences used for amplifying promoter region of *PLL *gene family members**. Click here for file

## References

[B1] Caffall KH Mohnen D The structure, function, and biosynthesis of plant cell wall pectic polysaccharides Carbohydr Res 2009 344 1879 1900 10.1016/j.carres.2009.05.021 19616198

[B2] Ridley BL O'Neill MA Mohnen D Pectins: structure, biosynthesis, and oligogalacturonide-related signaling Phytochemistry 2001 57 929 967 10.1016/S0031-9422(01)00113-3 11423142

[B3] Willats WG McCarney L Mackie W Knox PJ Pectin: cell biology and prospects for functional analysis Plant Mol Biol 2001 47 9 27 10.1023/A:1010662911148 11554482

[B4] Iwai H Masaoka N Ishii T Satoh S A pectin glucuronyltransferase gene is essential for intercellular attachment in the plant meristem Proc Natl Acad Sci USA 2002 99 16319 16324 10.1073/pnas.252530499 12451175 PMC138609

[B5] Jarvis M Briggs S Knox J Intercellular adhesion and cell separation in plants Plant Cell Enviro 2003 26 977 989 10.1046/j.1365-3040.2003.01034.x

[B6] Knox JP Seymour GB, Knox JP Cell and developmental biology of pectins Pectins and Their Manipulation 2002 CRC press LLC 131 146

[B7] Barras F van Gijsegem F Chatterjee AK Extracellular enzymes and pathogenesis of soft-rot *Erwinia* Annu Rev Phyto 1994 32 201 234

[B8] Collmer A Keen N The role of pectic enzymes in plant pathogenesis Annu Rev Phytopathol 1986 24 383 409 10.1146/annurev.py.24.090186.002123

[B9] Kotoujansky A Molecular genetics of pathogenesis by soft rot Erwinias Annu Rev Phytopathol 1987 25 405 430 10.1146/annurev.py.25.090187.002201

[B10] De Lorenzo G Cervone F Hahn MG Darvill A Albersheim P Bacterial endopectate lyase: evidence that plant cell wall pH prevents tissue maceration and increases the half-life of elicitor-active oligogalacturonides Physiol Mol Plant Pathol 1991 39 335 344 10.1016/0885-5765(91)90015-A

[B11] Fagard M Dellagi A Roux C Périno C Rigault M Boucher V Shevchik V Expert D *Arabidopsis thaliana *expresses multiple lines of defense to counterattack Erwinia chrysanthemi Mol Plant Microbe Interact 2007 20 794 805 10.1094/MPMI-20-7-0794 17601167

[B12] Norman-Setterblad C Vidal S Palva ET Interacting signal pathways control defense gene expression in *A. thaliana *in response to cell wall-degrading enzymes from *Erwinia carotovora* Mol Plant Microbe Interact 2000 13 430 438 10.1094/MPMI.2000.13.4.430 10755306

[B13] Wing R Yamaguchi J Larabell S Ursin V McCormic S Molecular and genetic characterization of two pollen-expressed genes that have sequence similarity to pectate lyases of the plant pathogen *Erwinia* Plant Mol Biol 1990 14 17 28 10.1007/BF00015651 1983191

[B14] Marín-Rodríguez MC Orchard J Seymour GB Pectate lyases, cell wall degradation and fruit softening J Exp Bot 2002 53 2115 2119 10.1093/jxb/erf089 12324535

[B15] Henrissat B Coutinho PM Davies GJ A census of carbohydrate-active enzymes in the genome of *Arabidopsis thaliana* Plant Mol Biol 2001 47 55 72 10.1023/A:1010667012056 11554480

[B16] Palusa SG Golovkin M Shin SB Richardson DN Reddy AS Organ-specific, developmental, hormonal and stress regulation of expression of putative pectate lyase genes in Arabidopsis New Phytol 2007 174 537 550 10.1111/j.1469-8137.2007.02033.x 17447910

[B17] Zhang Z Evolution by gene duplication: an update Trends Ecol Evol 2003 18 292 298 10.1016/S0169-5347(03)00033-8

[B18] Lynch M Force A The probability of duplicate gene preservation by subfunctionalization Genetics 2000 154 459 473 10629003 10.1093/genetics/154.1.459 PMC1460895

[B19] Benitéz-Burraco A Blanco-Portales R Redondo-Nevado J Bellido ML Moyano E Caballero JL Muñoz-Blanco J Cloning and characterization of two ripening-related strawberry (*Fragaria × ananassa *cv. Chandler) pectate lyase genes J Exp Bot 2003 54 633 645 10.1093/jxb/erg065 12554706

[B20] Chourasia A Sane VA Nath P Differential expression of pectate lyase during ethylene-induced postharvest softening of mango (*Mangifera indica *var. Dashehari) Physiol Plant 2006 128 546 555 10.1111/j.1399-3054.2006.00752.x

[B21] Futamura N Kouchi H Shinohara K A gene for pectate lyase expressed in elongating and differentiating tissues of a Japanese willow (*Salix gilgiana*) J Plant Physiol 2002 159 1123 1130 10.1078/0176-1617-00837

[B22] Marín-Rodríguez M Smith D Manning K Orchard J Seymour GB Pectate lyase gene expression and enzyme activity in ripening banana fruit Plant Mol Biol 2003 51 851 857 10.1023/A:1023057202847 12777045

[B23] Mita S Nagai Y Asai T Isolation of cDNA clones corresponding to genes differentially expressed in pericarp of mume (*Prunus mume*) in response to ripening, ethylene and wounding signals Physiol Plant 2006 128 531 545 10.1111/j.1399-3054.2006.00749.x

[B24] Pua E Ong C Liu P Liu J Isolation and expression of two pectate lyase genes during fruit ripening of banana (*Musa acuminata*) Physiol Plant 2001 113 92 99 10.1034/j.1399-3054.2001.1130113.x

[B25] Domingo C Roberts K Stacey NJ Connerton I Ruiz-Teran F McCann M A pectate lyase from *Zinnia elegans *is auxin inducible Plant J 1998 13 17 28 10.1046/j.1365-313X.1998.00002.x 9680962

[B26] Laskowski M Biller S Stanley K Kajstura T Prusty R Expression profiling of auxin-treated Arabidopsis roots: toward a molecular analysis of lateral root emergence Plant Cell Physiol 2006 47 788 792 10.1093/pcp/pcj043 16621846

[B27] Milioni D Sado PE Stacey NJ Domingo C Roberts K McCann MC Differential expression of cell-wall-related genes during the formation of tracheary elements in the Zinnia mesophyll cell system Plant Mol Biol 2001 47 221 238 10.1023/A:1010647902487 11554474

[B28] Vogel JP Raab T Somerville C *PMR6*, a pectate lyase-like gene required for powdery mildew susceptibility in Arabidopsis Plant Cell 2002 14 2095 2106 10.1105/tpc.003509 12215508 PMC150758

[B29] Cai S Lashbrook CC Stamen abscission zone transcriptome profiling reveals new candidates for abscission control: enhanced retention of floral organs in transgenic plants overexpressing Arabidopsis ZINC FINGER PROTEIN2 Plant Physiol 2008 146 1305 1321 10.1104/pp.107.110908 18192438 PMC2259061

[B30] Swarup K Benková E Swarup R Casimiro I Péret B Yang Y Parry G Nielsen E Smet ID Vanneste S Levesque MP Carrier D James N Calvo V Ljung K Kramer E Roberts R Graham N Marillonnet S Patel K Jones JDG Taylor CG Schachtman DP Sandberg G Benfey P Friml J Kerr I Beeckman T Laplaze L Bennett MJ The auxin influx carrier LAX3 promotes lateral root emergence Nat Cell Biol 2008 10 946 954 10.1038/ncb1754 18622388

[B31] Jiménez-Bermúdez S Redondo-Nevado J Muñoz-Blanco J Caballero JL López-Aranda JM Valpuesta V Pliego-Alfaro F Quesada MA Mercado JA Manipulation of strawberry fruit softening by antisense expression of a pectate lyase gene Plant Physiol 2002 128 751 759 10.1104/pp.010671 11842178 PMC148936

[B32] Leslie ME Lewis MW Liljegren SJ Roberts JA, Gonzalez-Carranza Z Organ abscission Annual Plant Reviews 2007 25 Oxford: Blackwell Publishing Ltd 91 106

[B33] Ostergaard L Borkhardt B Ulvskov P Roberts JA, Gonzalez-Carranza Z Dehiscence Annual Plant Reviews 2007 25 Oxford: Blackwell Publishing Ltd 137 159

[B34] Jarvis M Intercellular separation forces generated by intracellular pressure Plant Cell Enviro 1998 21 1307 1310 10.1046/j.1365-3040.1998.00363.x

[B35] Roy S Jauneau A Vian B Analytical detection of calcium-ions and immunocytochemical visualization of homogalacturonic sequences in the ripe cherry tomato Plant Physiol Biochem 1994 32 633 640

[B36] Hallett IC MacRae EA Wegrzyn TF Changes in kiwifruit cell-wall ultrastructure and cell packing during postharvest ripening Int J Plant Sci 1992 153 49 60 10.1086/297006

[B37] Wen F Laskowski M Hawes M Roberts JA, Gonzalez-Carranza Z Cell separation in roots Annual Plant Reviews 2007 25 Oxford: Blackwell Publishing Ltd 91 106

[B38] Mollet JC Faugeron C Morvan H Roberts JA, Gonzalez-Carranza Z Cell adhesion, separation and guidance in compatible plant reproduction Annual Plant Reviews 2007 25 Oxford: Blackwell Publishing Ltd 69 90

[B39] Patterson SE Cutting loose. Abscission and dehiscence in Arabidopsis Plant Physiol 2001 126 494 500 10.1104/pp.126.2.494 11402180 PMC1540116

[B40] Smyth DR Bowman JL Meyerowitz EM Early flower development in Arabidopsis Plant Cell 1990 2 755 767 10.1105/tpc.2.8.755 2152125 PMC159928

[B41] Ferrándiz C Regulation of fruit dehiscence in Arabidopsis J Exp Bot 2002 53 2031 2038 10.1093/jxb/erf082 12324527

[B42] Liu PP Koizuka N Homrichhausen TM Hewitt JR Martin RC Nonogaki H Large-scale screening of Arabidopsis enhancer-trap lines for seed germination-associated genes Plant J 2005 41 936 944 10.1111/j.1365-313X.2005.02347.x 15743455

[B43] Malamy JE Benfey PN Organization and cell differentiation in lateral roots of *Arabidopsis thaliana.* Development 1997 124 33 44 9006065 10.1242/dev.124.1.33

[B44] Esau K Hydathodes Anatomy of Seed Plants 1977 New York: John Wiley & Sons 205 206

[B45] Aloni R Schwalm K Langhans M Ullrich CI Gradual shifts in sites of free-auxin production during leaf-primordium development and their role in vascular differentiation and leaf morphogenesis in Arabidopsis Planta 2003 216 841 853 12624772 10.1007/s00425-002-0937-8

[B46] Cheng Y Dai X Zhao Y Auxin synthesized by the YUCCA flavin monooxygenases is essential for embryogenesis and leaf formation in Arabidopsis Plant Cell 2007 19 2430 2439 10.1105/tpc.107.053009 17704214 PMC2002601

[B47] Berger F Endosperm: the crossroad of seed development Curr Opin Plant Biol 2003 6 42 50 10.1016/S1369526602000043 12495750

[B48] Schmid M Davison TS Henz SR Pape UJ Demar M Vingron M Schölkopf B Weigel D Lohmann JU A gene expression map of *Arabidopsis thaliana *development Nat Genet 2005 37 501 506 10.1038/ng1543 15806101

[B49] Stenvik GE Butenko MA Urbanowicz BR Rose JK Aalen RB Overexpression of INFLORESCENCE DEFICIENT IN ABSCISSION activates cell separation in vestigial abscission zones in Arabidopsis Plant Cell 2006 18 1467 1476 10.1105/tpc.106.042036 16679455 PMC1475485

[B50] Sabatini S Beis D Wokenfelt H Murfett J Guilfoyle T Malamy H Benfey P Leyser O Bechtold N Weisbeek P Scheres B An auxin-dependent distal organizer of pattern and polarity in the Arabidopsis root Cell 1999 99 463 472 10.1016/S0092-8674(00)81535-4 10589675

[B51] González-Carranza ZH Elliott KA Roberts JA Expression of polygalacturonases and evidence to support their role during cell separation processes in *Arabidopsis thaliana* J Exp Bot 2007 58 3719 3730 10.1093/jxb/erm222 17928369

[B52] The Arabidopsis Information Resource http://www.arabidopsis.org

[B53] Clustal: Multiple Sequence Alignment http://www.clustal.org

[B54] Larkin MA Blackshields G Brown NP Chenna R McGettigan PA McWilliam H Valentin F Wallace IM Wilm A Lopez R Thompson JD Gibson TJ Higgins DG Clustal W and Clustal x version 2.0 Bioinformatics 2007 23 2947 2948 10.1093/bioinformatics/btm404 17846036

[B55] Tamura K Dudley J Nei M Kumar S MEGA4: Molecular Evolutionary Genetics Analysis (MEGA) software version 4.0 Mol Biol Evol 2007 24 1596 1599 10.1093/molbev/msm092 17488738

[B56] Pfam Database http://pfam.sanger.ac.uk/

[B57] Finn RD Mistry J Tate J Coggill P Heger A Pollington JE Gavin OL Gunasekaran P Ceric G Forslund K Holm L Sonnhammer EL Eddy SR Bateman A The Pfam protein families database Nucleic Acids Res 2010 38 D211 22 10.1093/nar/gkp985 19920124 PMC2808889

[B58] SignalP 3.0 Server http://www.cbs.dtu.dk/services/SignalP/

[B59] Emanuelsson O Brunak S von Heijne G Nielsen H Locating proteins in the cell using TargetP, SignalP and related tools Nat Protoc 2007 2 953 971 10.1038/nprot.2007.131 17446895

[B60] National Center for Biotechnology Information http://www.ncbi.nlm.nih.gov

[B61] Clough SJ Bent AF Floral dip: a simplified method for Agrobacterium-mediated transformation *of Arabidopsis thaliana* Plant J 1998 16 735 743 10.1046/j.1365-313x.1998.00343.x 10069079

[B62] Eisen MB Spellman PT Brown PO Botstein D Cluster analysis and display of genome-wide expression patterns Proc Natl Acad Sci USA 1998 95 14863 14868 10.1073/pnas.95.25.14863 9843981 PMC24541

