# Supplementary figures and images for: Analysis of promoter activity of members of the PECTATE LYASE-LIKE (PLL) gene family in cell separation in Arabidopsis
Source: BMC Plant Biol. 2010 Jul 22;10:152. doi: 10.1186/1471-2229-10-152 (PMC3017822; doi:10.1186/1471-2229-10-152)

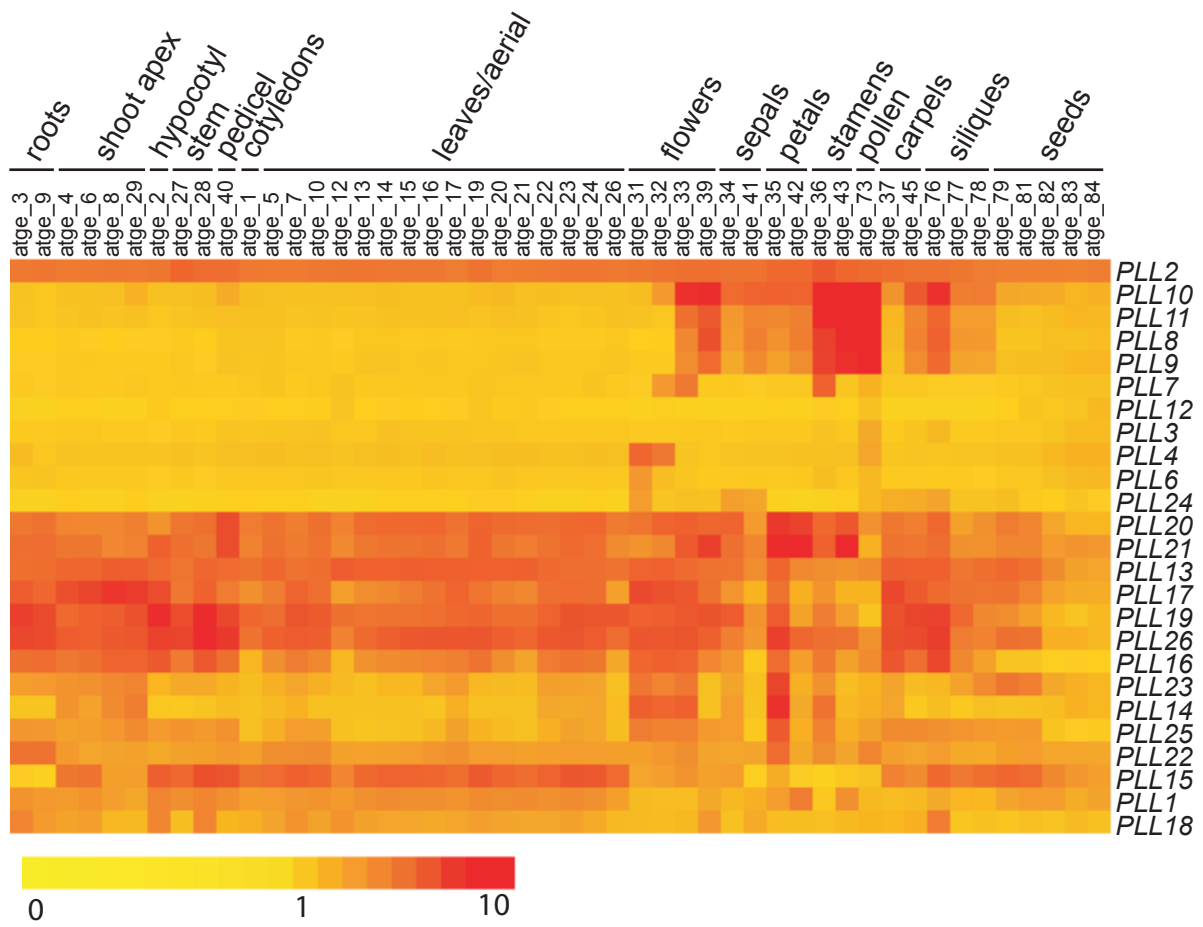

Supplement: Additional file 1 — Hierarchical clustering of RNA accumulation for Arabidopsis PLL genes in various plant parts/developmental stages based on public microarray data. Plant parts and growth stages are labeled according to AtGenExpress [48]. Microarray signal value is indicated by color from yellow (lowest) to red (highest). PLL5 was not represented on the microarray used in this analysis. [file 1471-2229-10-152-S1.PDF]

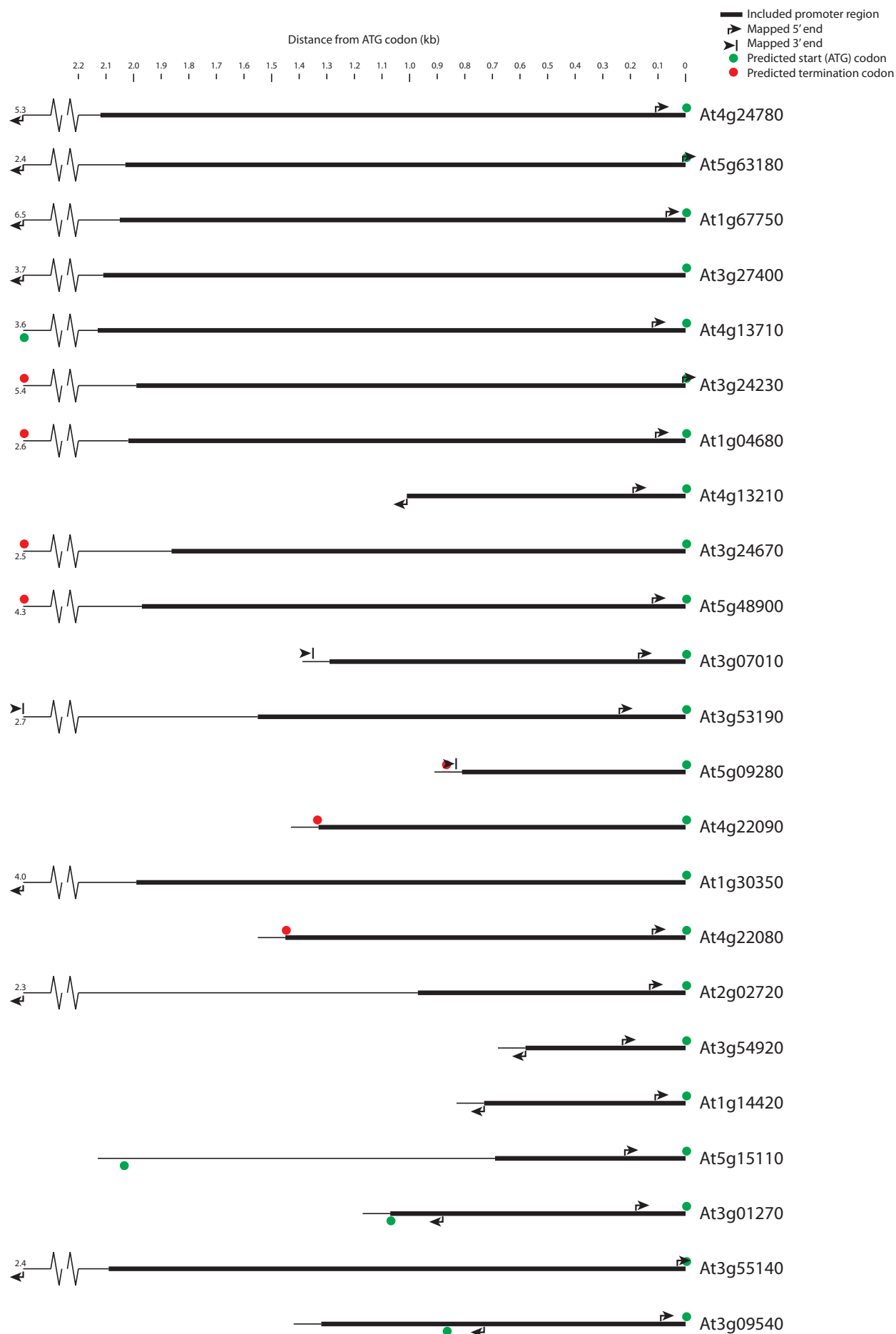

Supplement: Additional file 2 — Depiction of promoter region from individual PEL genes utilized to drive GUS expression. Genes are aligned with the predicted translational start (ATG) codon at right. The scale of upstream distance (kb) is given at top. The portion of the upstream region used is indicated with a heavy line. Upstream features (mapped 5' or 3' RNA ends and/or predicted translational start/stop codons) delineating the intergenic region are indicated with relative position given at left. [file 1471-2229-10-152-S2.PDF]
