# Supplementary material for: Analysis of promoter activity of members of the PECTATE LYASE-LIKE (PLL) gene family in cell separation in Arabidopsis
Source: BMC Plant Biol. 2010 Jul 22;10:152. doi: 10.1186/1471-2229-10-152 (PMC3017822; doi:10.1186/1471-2229-10-152)
Supplement: Additional file 4 — Primer sequences used for amplifying promoter region of PLL gene family members. [file 1471-2229-10-152-S4.PDF]

At4g24780	5' -AACCATGGTTCTCTCTCTCTCTCACTTGG- 3'<br>5’ -ATAAGCTTCCACCTATCATTACCAACACC- 3'
At5g63180	5' -TTCCATGGATGAGTGAAGAGAGA AAGACA-3'<br>5' -CGAAGCTTTAACACTGATAAATGAGTAGA- 3'
At1g67750	5' -AACCATGGTCTTGTCTCTCGAGAGGATT- 3'<br>5' -CGAAGCTTTATCAATGTGTATCATGAAT- 3'
At3g27400	5' -ACCATGGTCTTCTAAACATAGATTGAGA- 3'<br>5' -CGGAATTCGCAAATGGCACTATAAACCAC- 3'
At4g13710	5' -AACCATGGTGAAGCTTTCTTCTTCTTCT- 3'<br>5' -AAGAATTCTGCACAAGAGACATAAAAGT- 3'
At3g24230	5' -AACCATGGTGTGCAAACAAAGGGAAAATG- 3'<br>5' -ATGAATTCACGAAAAATATAGCGTGACGG- 3'
At1g04680	5' -AACCATGGTGGAGAGGCAGAAGCTGAGCC- 3'<br>5' -AAGGTACCCACTTCAAGTCTTCGAAAGTA- 3'
At4g13210	5' -AACCATGGTGTTGGTTGTTGTTAGAGTT- 3'<br>5' -TTGGTACCGCCGAAACAATAACCTCTTT- 3'
At3g24670	5' -AACCATGGTGTTGGATATATCAAAGCTCT- 3'<br>5' -AAGGTACCCAGGGTGCTTGTAAATTATGT- 3'
At5g48900	5' -AACCATGGTGTTCTTGCTCTGTTCTGTT- 3'<br>5' -AAGGTACCAAATCATGTTTTCCCGCCAA- 3'
At3g07010	5' -AACCATGGTGTGACAGCCATTGTTATGGC-3'<br>5' -AAGGTACCCTCGTAAGTTCCTTACCTATG3'
At3g53190	5' -AACCATGGTGCTGAAGAAACTTGTGATT- 3'<br>5' -GAAAGCTTAATCAGTAACTTTATTGACA- 3'
At5g09280	5' -AACCATGGTTTTCCGGCAAATCCGACTGA- 3'<br>5' -AAGGTACCAGTTTATTCAGGTCATGTGT- 3'
At4g22090	5' -AACCATGGTAGTAATGTTGCATTTACTT- 3'<br>5' -AAGGTACCATGAGGCAGCTGCCACCCTT- 3'
At1g30350	5' -CGCCATGGTTTCTTGAAAATGTGATGCT- 3'<br>5' -AAGGATCCCCAAAGCCTTTTGCTGATA- 3'
At4g22080	5' -AACCATGGGGTTGGGTGGGTTTATGGTT- 3'<br>5' -ATAAGCTTTATTCAATACTCTTTTCACG- 3'
At2g02720	5' -AACCATGGTTGAATCAGCAGTGGTGAGA- 3'<br>5' -ATAAGCTTGATTGCGATCATCAAAAGTT- 3'
At3g54920	5' -AACCATGGCGTTAGTGGCGGATTTTGAC- 3'<br>5' -ACAAGCTTTCAGAAGATACCACAATCGC- 3'
At1g14420	5' -TACCATGGTTTAAATATATTGCAAATGC- 3'<br>5' -ATAAGCTTTTAATTACTTGTATGATAAT- 3'
At5g15110	5' -GCCCATGGCTTTCTTTTTTTGTTTTCAA- 3'<br>5' -CAGGATCCAAGATATATATAATAGTCTC -3'
At3g01270	5' -AACCATGGTTTATTTGATTACCCCTTTC- 3'<br>5' -TTGGATCCTGTTGCTTATCTGAGAAAGT- 3'
At3g55140	5' -AACCATGGCGTTCGTTGTTATGCGACGT- 3'<br>5' -ACAAGCTTTCTTGTACATACAGCAGAGA- 3'
At3g09540	5’ -GGCCATGGATCTAATTTATATAACAAATG- 3'<br>5' -ATAAGCTTCCATGTTTAACATACTTCTT- 3'
